# Anticancer Activity of Saponins from *Allium chinense* against the B16 Melanoma and 4T1 Breast Carcinoma Cell

**DOI:** 10.1155/2015/725023

**Published:** 2015-06-03

**Authors:** Zhihui Yu, Tong Zhang, Fengjuan Zhou, Xiuqing Xiao, Xuezhi Ding, Hao He, Jie Rang, Meifang Quan, Ting Wang, Mingxing Zuo, Liqiu Xia

**Affiliations:** Hunan Provincial Key Laboratory of Microbial Molecular Biology-State Key Laboratory Breeding Base of Microbial Molecular Biology, College of Life Science, Hunan Normal University, Changsha 410081, China

## Abstract

The cytotoxic substance of *A. chinense* saponins (ACSs) was isolated using ethanol extraction and purified with the D_101_ macroporous adsorption resin approach. We investigated the anticancer activity of ACSs in the B16 melanoma and 4T1 breast carcinoma cell lines. Methylthioninium chloride and hematoxylin-eosin staining with Giemsa dyestuff were used when the cells were treated with ACSs. The results showed that the cells morphologies changed significantly; ACSs induced cell death in B16 and 4T1 cells based on acridine orange/ethidium bromide double fluorescence staining, with the number and degree of apoptotic tumor cells increasing as ACS concentration increased. ACSs inhibited the proliferation of B16 and 4T1 cells in a dose-dependent manner. They also inhibited cell migration and colony formation and exhibited a concentration-dependent effect. In addition, ACSs apparently inhibited the growth of melanoma in vivo. The preliminary antitumor in vivo assay revealed that early medication positively affected tumor inhibition action and effectively protected the liver and spleen of C57 BL/6 mice from injury. This study provides evidence for the cytotoxicity of ACSs and a strong foundation for further research to establish the theoretical basis for cell death and help in the design and development of new anticancer drugs.

## 1. Introduction

Patients with cancer generally undergo surgical therapy, chemotherapy, radiotherapy, or a combination of these treatments. Although the effects of these treatments are significant, it is a fact that most patients suffer from side effects, such as general fatigue, high fever, loss of appetite, and many kinds of infections. In Japan and China, herbal medicine remedies, including* Allium* species, ginger, and ginseng, are used for the supplemental treatment of various cardiovascular diseases [1, 2], hypertension [3], diabetes [4], Alzheimer's disease [5, 6], inflammation, and thrombosis [7]. Recent research has shown that the use of herbal remedies for cancer treatment resulted in fewer or diminished side effects induced by Western medicine, such as chemotherapy and radiotherapy, and a longer survival period for many patients [8, 9]. Herbal medicine has also been reported to be able to prevent the progression of colon carcinoma, gastric cancer, and breast cancer, as well as their metastasis to the liver, lung, and bone. Moreover, hepatocellular carcinoma has been shown to become smaller without severe side effects after treatment with herbal medicine [10]. Although these reports strongly suggest that herbal medicine remedies would be good candidates for the treatment of several types of cancer, the mechanisms by which they could improve the clinical status, including cancer metastasis, of cancer patients remain unclear.

Many active substances in herbs have good antitumor activity, such as flavone [11, 12], polyphenol [13, 14], paclitaxel [15, 16], and camptothecin [17, 18]. These agents are present in the diet as a group of compounds with low toxicity that are safe and generally accepted [19]. Saponins isolated from different plants are considered indicators in a number of cancer cell lines. The induction of apoptosis by saponins has been described in various studies [20–23]. Several tests and research results have demonstrated the antitumor properties of saponins, including inhibition of cancer cell proliferation [24, 25] and migration [26, 27]. The main effective ingredients of Chinese herbal medicine remedies, such as ginseng,* Polygala tenuifolia*, licorice, and Radix Bupleuri, among others, are saponin materials. Many saponin materials have been reported to exhibit cytotoxic effects on the B16 [28] and 4T1 [29] cell lines and induce apoptosis. A number of studies and clinical application products have proven that saponin materials achieve an effective dose and have no side effects on the human body, suggesting that they may have potential antitumor activity.

Plants of the genus* Allium* in the lily family are well known in the Northern Hemisphere [30], and many of them are being used as traditional medicine and have been found to be effective in the treatment of several diseases [31].* Allium* species are generally used as culinary spices in Asia, especially in China. Saponins isolated from the genus* Allium* have been shown to inhibit the growth of such human cancer cell lines as the PC12 [32], 3T3-L1 [33], MCF-7, NCI-H460, SF-268, HepG2 [34], human colorectal cancer [35], and HeLa [36] cell lines. Similar results had also been reported by other studies on ethanol extracts, such as isolated polyphenols and green tea polyphenols [37, 38]. Oświecimska et al. reported that saponin fractions and extracts from* Primula* species have antimitotic activity [39]. In recent years, research on the anticarcinogenic potential of saponins has shown that they are promising anticancer agents. Likewise, in vitro and in vivo studies have shown that saponins are able to inhibit the viability of a variety of malignant tumor cells. Mimaki et al. reported on the inhibitory activity of steroidal saponins from the bulbs of* Triteleia lactea* on cyclic AMP phosphodiesterase [40]. Kuroda et al. also reported that steroidal saponins from* Allium chinense* (jiao tou) had inhibitory action on cyclic AMP phosphodiesterase and Na^+^/K^+^ ATPase [41]. Peng et al. obtained six compounds isolated from fresh bulbs of garlic. Their structures were elucidated as proto-iso-eruboside-B, eruboside-B, and iso-eruboside-Band found to affect platelet aggregation, blood coagulation, and fibrinolysis in vitro [42]. Sata et al. investigated steroidal saponins from the bulbs of an elephant garlic mutant and determined that they had an antifungal function [43]. Saponin materials also exhibit antibacterial and antipyretic activities, produce a calming effect, increase DNA and protein synthesis, and improve the organism's immune ability, among their other physical and pharmacological functions.

It is important to explore the cell death of* A. chinense* (jiao tou) saponins (ACSs) as a novel therapeutic agent. The objectives of this study were to isolate major ACSs and evaluate their anticancer effects on B16 and 4T1 cells, which are widely used in in vitro and in vivo models of anticancer function. Our findings provide the necessary theoretical framework and technical guidance for the antitumor study of ACSs.

## 2. Materials and Methods

### 2.1. Reagents

ACSs were extracted with ethanol, dissolved in phosphate buffered saline (PBS) (pH 7.8; China), and diluted in cell culture medium for preparation. Dulbecco's modified Eagle's medium (DMEM), fetal bovine serum (FBS), 2 mmol/L l-glutamine, 100 U/mL penicillin, and 100 mg/mL streptomycin were purchased from Gibco Life Technologies (Grand Island, USA). Giemsa and hematoxylin-eosin (HE) stains were purchased from Ameresco (USA). 5-Fluorouracil (5-Fu), l-Dopa, bovine serum albumin, and phenylmethanesulfonyl fluoride (PMSF) were purchased from BBI (USA). Acridine orange (AO), ethidium bromide (EB), dimethyl sulfoxide, trypan blue, trypsin, and ethylenediaminetetraacetic acid were purchased from Beijing Dingguo Changsheng Biotech Co., LTD. (China). Triton X-100 and Coomassie Brilliant Blue G_250_ were obtained from Shanghai Sangon Biological (China). Six orifice plates and tissue culture flasks were purchased from NUNC Company (Denmark). Other chemicals were purchased from local companies. Solutions were prepared using a Milli-Q ultrapure water system (Millipore Corp.).

### 2.2. Extraction and Purification of ACSs

Two-year-old bulbs of* A. chinense* (jiao tou) were obtained from the XiangYin* A. chinense* (jiao tou) export base in Hunan Province. Specimens were identified as described in the literature and according to related research on traditional Chinese medicine. The bulbs of* A. chinense* (jiao tou) were dried, crushed, and screened via a 200-mesh screen and then extracted with 60% ethanol at room temperature to obtain a crude extract. The crude extract was dissolved in water and extracted successively with petroleum ether and* n*-butanol. The* n*-Butanol extract was evaporated under reduced pressure conditions in a rotavapor. The residue was redissolved in 70% ethanol and purified with D_101_ macroporous adsorption resin, enriched, and dried, after which the ACSs were obtained.

### 2.3. Cell Lines and Cell Culture

The B16 and 4T1 cell lines were obtained from our laboratory. All cells were cultured in DMEM supplemented with 10% FBS, 2 mM l-glutamine, 100 U/mL penicillin, and streptomycin. Cells were incubated in a constant-temperature incubator under a humidified atmosphere containing 5.0% CO_2_ at 37°C. Stock cultures were subcultured every 2-3 days using 0.25% trypsin and 0.02% ethylenediaminetetraacetic acid. The cells were incubated for 24 h and treated with different concentrations of ACSs.

### 2.4. Cell Death-Induced Morphological Changes in Cells

Cell morphological changes were observed directly under an inverted phase contrast microscope (Leika, Co., Ltd., Germany) and with oil immersion lens (Olympus, Co., Ltd., Tokyo, Japan) after Giemsa and HE staining. The cells then underwent logarithmic proliferation and mixed with PBS, 5-Fu, and ACSs (100, 200, and 300 *μ*g/mL) for 24 h. A control group was established at the same time. Giemsa and HE dyestuff were used to stain the cells, all of which were cleaned with PBS twice (5 min each time), fixed in formaldehyde for 10 min, mixed with Giemsa or HE dye liquor for 10 min, and then cleaned by removing excess liquid after fragrant asphalt was added. After HE staining, the samples were dried and sealed with neutral gum.

### 2.5. AO/EB Double Fluorescence Staining

B16 and 4T1 cells were treated with ACSs, after which the cultures were removed and washed with PBS twice (5 min each time), fixed in 95% ethanol for 10 min, mixed with 0.1 *μ*g/mL of AO and EB for 10 min, and washed with PBS twice (5 min each time). Finally, a fluorescence-quenching seal tablet was added to the mixture. Cell death was evaluated under laser scanning confocal microscope (Zeiss Co., Germany).

### 2.6. Trypan Blue Exclusion Assay

B16 and 4T1 cells were cultured at a density of 1 × 10^6^ cells/mL in 60-mm culture dishes. After 24 h of attachment, all cells were treated with ACSs. After 6–96 h, the cells were harvested and washed with PBS twice; the cell pellets were resuspended in PBS. After incubation in 0.4% trypan blue for 5 min (maximum: 10 min), viable cells were counted using a hemocytometer. At indicated time points before they were harvested, the cells were visualized under an inverted phase contrast microscope to observe changes in their proliferation and the number of living cells upon treatment was counted.

### 2.7. Cell Migration Rate Assay

Cell scarification is one of the best methods to determine the motion characteristics of tumor cells. Under an in vitro injury-healing experimental model, cells were cultured in six orifice plates, after which the cell layers were scratched, cultured in vitro, and subjected to injury. Cells were washed three times with medium to remove detached cells. All cells were treated with ACSs. Finally, a culture medium without newborn calf serum was used to reduce the influence of cell proliferation after treatment for 24 h. The wound area in the microscopic images was measured using Image J software. The percentage of wound healing after 24 h was calculated relative to the total wound area at 0 h of the same wound spot.

### 2.8. Cell Colony Formation Assay

When a single cell proliferates to six generations or more in vitro, its offspring becomes a colony each containing more than 50 cells. The colony formation rate indicates the independent viability of cells. Logarithmic phase cells were collected, and their cell concentrations were modulated. Approximately 100 cells were placed in each culture dish, adhered to the wall, were cut into sections, and were treated with ACSs. The vaccination was repeated three times, and the cells were treated with drugs for 7 days, fixed in 90% methanol, stained with Giemsa liquid dye for 5 min, and cleared with xylene. The cells were observed and counted under an inverted microscope (50 cells are equivalent to one colony), and the cell colony formation and inhibition rates were calculated as follows:

(1) Rcf=NcfNvc×100%,RIcf=RTcfRCcf×100%,

where *R*_cf_ is the rate of colony formation, *N*_cf_ is the number of colonies formed, *N*_vc_ is the number of vaccination cells, *R*_Icf_ is the rate of colony formation inhibition, *R*_Tcf_ is the colony formation rate in the treatment group, and *R*_Ccf_ is the colony formation rate in the control group.

### 2.9. Tyrosinase Activity Assay

A source of crude cellular tyrosinase was obtained by homogenizing drug-treated or untreated cells in 20 mM sodium phosphate (pH 6.8), 1% Triton X-100, and 1 mM PMSF at 4°C with 30 repeated strokes to determine the tyrosinase activity in the crude extract. Detergent was used to release the membrane-bound tyrosinase from the melanosomes. The lysates were centrifuged at 15,000 rpm for 15 min to obtain the supernatant as the source of crude cellular tyrosinase. The protein content in the supernatant was determined using Bradford assay with bovine serum albumin as the protein standard. Tyrosinase activity was then determined as follows: 1 mL of the reaction mixture containing 50 mM PBS, 2.5 mM l-Dopa, and 500 *μ*g of the supernatant protein was incubated at 37°C for 15 min, following which dopachrome formation was monitored by measuring absorbance at the wavelength of 475 nm. One unit of tyrosinase activity was defined as the total enzyme that catalyzes the formation of 1 *μ*mol of dopachrome in 1 min. The amount of dopachrome in the reaction was calculated using the Lambert-Beer Law, whereas the molar extinction coefficient of dopachrome was 3600 M^−1^·cm^−1^ [44]. Specific tyrosinase activity was normalized with protein content in the reaction. Untreated or ACS-treated cells were harvested, washed with PBS twice, and lysed in 20 mM sodium phosphate and 1 mM PMSF at 4°C in the absence of detergent with 50 strokes to prepare a melanosome-enriched fraction. The lysate was centrifuged at 1000 g for 15 min at 4°C to pellet the nuclei and unbroken cells, and the resulting supernatant was centrifuged at 40,000 g for 30 min at 4°C to obtain a melanosome-enriched pellet. The pellet was then resuspended in 0.5 mL of 20 mM sodium phosphate containing 1 mM PMSF and used as the enzyme source. The kinetics of melanosome-enriched tyrosinase activity was conducted in a 1 mL reaction mixture containing 50 mM PBS, 2.5 mM l-Dopa, and 70 *μ*g of the supernatant protein from the melanosome-enriched enzyme source. Dopachrome formation of the reaction mixture was kinetically monitored by measuring absorbance at the wavelength of 475 nm every minute at 37°C.

### 2.10. Animal Model and Drug Treatment

We conducted our experiment for a chemotherapy drug using C57 BL/6 mice after receiving approval from the animal care and use committee of our institution. Male C57 BL/6 mice (4–6 weeks old) were obtained from Xiangya Medical College (Changsha, Hunan) and maintained in a pathogen-free environment. Eight mice were housed per cage, fed animal food, and observed daily. The cages were kept in a climate-controlled, warm animal suite and cleaned on a weekly basis.

The effects of in vivo tumor development were induced in C57 BL/6 mice by the inoculation of B16 cells in two routes. In the first model, B16 cells (5 × 10^5^) were inoculated subcutaneously. The anticancer drugs were used immediately the following day. Chemotherapy was administered every day for 18 days. In the second model, B16 melanoma cells (5 × 10^5^) in 100 *μ*L of PBS were injected directly via subcutaneous administration. At 1 week after B16 melanoma cell injection, the mice were injected with 100 *μ*L of the chemotherapy drug by intraperitoneal injection, as described previously [45]. For each model, chemotherapy treatment of C57BL/6 mice was randomized into six groups of eight mice each. The control group was injected without any materials. Of the five single-drug treatment groups, four groups were injected with ACSs (low-dose group: 50 mg/kg body weight; high-dose group: 200 mg/kg body weight) and one group was injected with PBS [46]. The mice were treated with the chemotherapy drug every day for 18 days (ACSs were injected on the following day) and 11 days (ACSs were injected after 1 week). The mice were closely monitored. They were weighed every day, and the length and width of the tumors were measured with a vernier caliper every day as well. After the tumor had grown to a certain volume, the mice were killed by spinal dislocation; several tissues (tumor, spleen, and liver) were removed and measured using the tumor size calculation formula, *V*_*t*_ = (*L* × *W* ^2^)/2. The tumor, spleen, and liver indices were also calculated as follows:

(2) Tumor  index  %=Tumor  weight  mgBody  weight  g×100%,Spleen  index  %=Spleen  weight  mgBody  weight  g×100%,Liver  index  %=Liver  weight  mgBody  weight  g×100%.

### 2.11. Statistical Analysis

Statistical analysis was performed using SPSS. One-way ANOVA with Fisher's least significant difference was used to analyze variations, whereas ANOVA was used to determine the significance of relationships. *P* < 0.05 was considered statistically significant.

## 3. Results

### 3.1. ACSs Induced Morphological Changes

**Figure 1:**
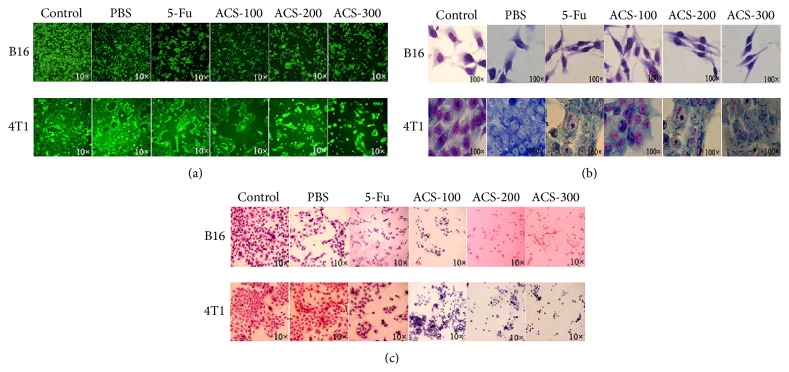
Effects of ACS treatment on the morphologies of B16 and 4T1 cells. B16 and 4T1 cells were treated with different concentrations of ACSs (100, 200, and 300 *μ*g/mL). The cells were divided into three groups: the positive group (5-Fu; 30 *μ*g/mL), the negative group (PBS; 100 *μ*L), and the control group. (a) Representative images showing the morphological changes in B16 and 4T1 cells after being treated with different concentrations of ACSs (original magnification ×10). (b) Representative images showing the morphological changes in B16 and 4T1 cells dyed with Giemsa after being treated with different concentrations of ACSs (original magnification, ×100). (c) Representative images showing the morphological changes in B16 and 4T1 cells dyed with HE after being treated with different concentrations of ACSs (original magnification, ×10).

ACSs had a significant effect on B16 and 4T1 cells. Left untreated, all cells were connected by spindle structures and in a stretched state; however, after being treated with 5-Fu or ACSs, they shrank and exhibited many small cell fragments (Figure 1(a)). After Giemsa staining, cells settled on the bottom of the dish and were connected closely to one another; the cell nuclei stained a lighter color. Small cellular tubules and microsilk were observed. The morphology of the cells that did not receive any materials or undergo PBS treatment did not exhibit any changes; in contrast, B16 and 4T1 cells subjected to treatment with ACSs or 5-Fu for 24 h displayed similar apoptotic activities, with elliptic or spherical cell spindles, pigmented cell nuclei, reduced adhesion, and several apoptotic bodies. In addition, 4T1 breast carcinoma cells produced many empty bubble structures (Figure 1(b)). All cells were dyed with HE, and the results showed that the originally spindle-shaped or polygonal B16 cells adopted an irregular form; the nuclei stained violet or blue-black and underwent solid shrinkage. 4T1 cells started floating, appeared cloudy or disrupted, and formed apoptotic bodies, among others (Figure 1(c)).

### 3.2. ACSs Accelerated Cell Death and Exhibited Concentration Dependence

**Figure 2:**
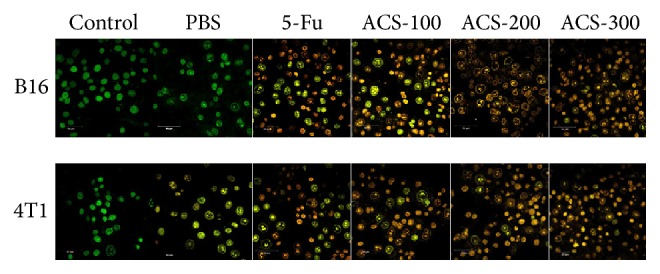
Apoptosis in B16 and 4T1 cells detected by AO/EB double fluorescence staining. The mitochondrial membrane potentials in B16 and 4T1 cells after being treated with different concentrations of ACSs (100, 200, and 300 *μ*g/mL) and 5-Fu (30 *μ*g/mL) for 24 h and then stained with AO and EB are illustrated. Representative images show the degree of ACS- and 5-Fu-induced Anticancer Activity in B16 and 4T1 cells. Treatment with PBS and the control conditions did not exhibit the same phenomenon. Original magnification, ×100.

B16 and 4T1 cells were stained with AO and EB to distinguish among the early apoptotic, late apoptotic, living, and necrotic cells.  Figure 4 shows that the living cells had normal structures and their nuclear chromatin stained green, the necrotic cells had normal structures and their nuclear chromatin stained red, the early apoptotic cells exhibited abnormal structures as well as a certain degree of shrinkage or a round pearl shape and their nuclear chromatin stained yellow, and the late apoptotic cells stained jacinth. Treatment with PBS apparently did not affect the degree of cell death in B16 and 4T1 cells, whereas treatment with 5-Fu and ACSs apparently did not influence necrosis. The degree of cell death and number of tumor cells increased with increasing ACS concentration (Figure 2).

### 3.3. ACSs Inhibited Cell Proliferation and Exhibited Dose Dependence

**Figure 3:**
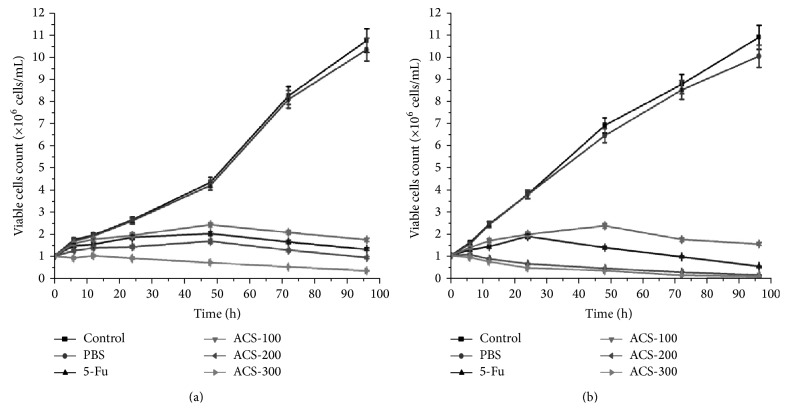
Effects of ACSs on the proliferation of B16 and 4T1 cells. B16 and 4T1 cells were treated with different concentrations of ACSs (100, 200, and 300 *μ*g/mL) and 5-Fu (30 *μ*g/mL) for 24 h and then dyed with Giemsa. Representative images show that ACSs inhibited the proliferation of B16 and 4T1 cells but that PBS and the control conditions did not.

Normal and healthy cells can reject trypan blue and remain colorless; however, apoptotic and dead cells can be stained, because death damages cell membrane integrity. When we used 5-Fu and ACSs to process B16 and 4T1 cells, we determined that ACSs effectively inhibited tumor cell proliferation. On the other hand, we found that ACSs inhibited tumor cell proliferation in a dose-dependent manner and exhibited negative growth, compared with blank control, but almost had no effect when treated with PBS (Figure 3).

### 3.4. ACSs Inhibited the Migration Rates of B16 and 4T1 Cells

**Figure 4:**
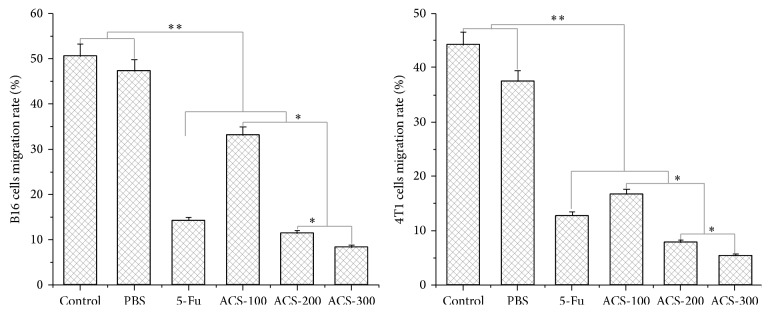
Migration rates of B16 and 4T1 cells treated with ACSs. B16 and 4T1 cells were cocultured with ACSs using DMEM without FBS to eliminate the influence of cell growth and proliferation. Cell migration rates were determined using cell scarification.

ACSs inhibited the migration ability of B16 and 4T1 cells, with their migration rates exhibiting a downward trend as the concentration of ACSs increased. The migration rates of B16 cells were 33.1%, 11.4%, and 8.3% at the ACS concentrations of 100, 200, and 300 *μ*g/mL, respectively. As for 4T1 cells, the migration rates at the corresponding ACS concentrations were 16.7%, 7.8%, and 5.4%, respectively. These results indicate that ACSs possessed excellent inhibitory effects on the migration of B16 and 4T1 cells but that their inhibitory effect on B16 cells was better than that on 4T1 cells. 5-Fu served as the clinical antitumor drug and resulted in inhibition rates of up to 14.2% and 12.8% for B16 and 4T1 cells, respectively. PBS exhibited weak activity in B16 cells but somewhat inhibited the migration of 4T1 cells, which could be attributed to the fact that PBS altered the salt concentration of the culture medium and prevented the cells from adapting to the situation within a short period, thereby inhibiting their migration (Figure 4).

### 3.5. ACSs Inhibited Cell Colony Formation and Exhibited Dose Dependence

**Figure 5:**
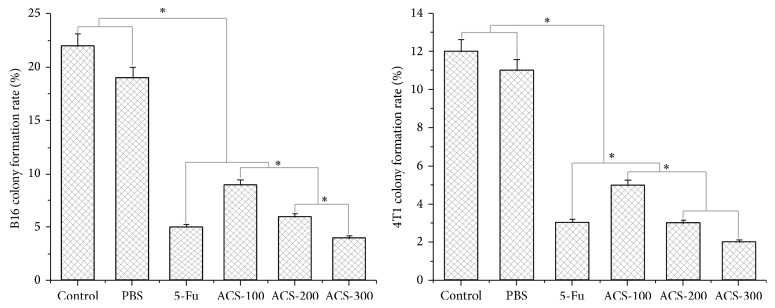
Colony formation rates of B16 and 4T1 apoptotic cells induced by ACSs. Cells were cocultured with drugs for 24 h, observed, and counted after Giemsa staining. Control: treatment without materials; PBS: negative control, 100 *μ*L/mL; 5-Fu: positive control, 30 *μ*g/mL; ACS-100: 100 *μ*g/mL; ACS-200: 200 *μ*g/mL; ACS-300: 300 *μ*g/mL.

**Figure 6:**
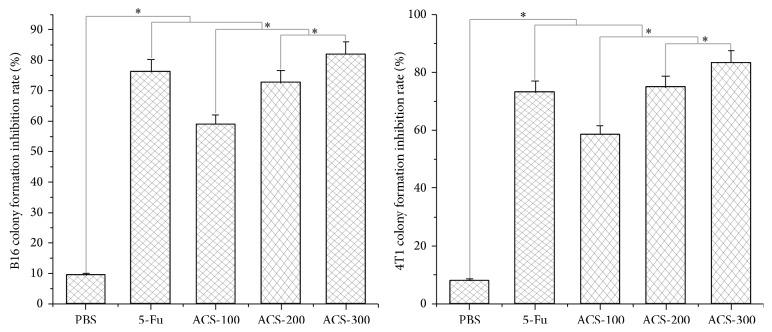
Colony formation inhibition rates of B16 and 4T1 cells induced by ACSs. Control: treatment without materials. PBS: negative control, 100 *μ*l/mL. 5-Fu: positive control, 30 *μ*g/mL. ACS-100: 100 *μ*g/mL. ACS-200: 200 *μ*g/mL. ACS-300: 300 *μ*g/mL.

ACSs have the ability to induce cell death in B16 and 4T1 cells, thereby affecting tumor cell proliferation and cell colony formation. Under the control conditions, the cell colony formation rates of B16 and 4T1 cells were 22% and 12%, respectively. Treatment with PBS did not exhibit any influence on cell colony formation and its inhibition in B16 and 4T1 cells, whereas treatment with 5-Fu and ACSs significantly affected them. Moreover, the rate of cell colony formation decreased gradually with increasing ACS concentration. The colony formation rates of B16 cells were 9%, 6%, and 4% at the ACS concentrations of 100, 200, and 300 *μ*g/mL, respectively; their corresponding colony formation inhibition rates were 59.1%, 72.8%, and 81.9%, respectively. The colony formation rates of 4T1 cells were 5%, 3%, and 2% at the ACS concentrations of 100, 200, and 300 *μ*g/mL, respectively; their corresponding colony formation inhibition rates were 58.4%, 75.0%, and 83.4%, respectively (Figures 5 and 6).

### 3.6. Inhibition of Tyrosinase Activity of B16 Cells Treated with ACSs

**Figure 7:**
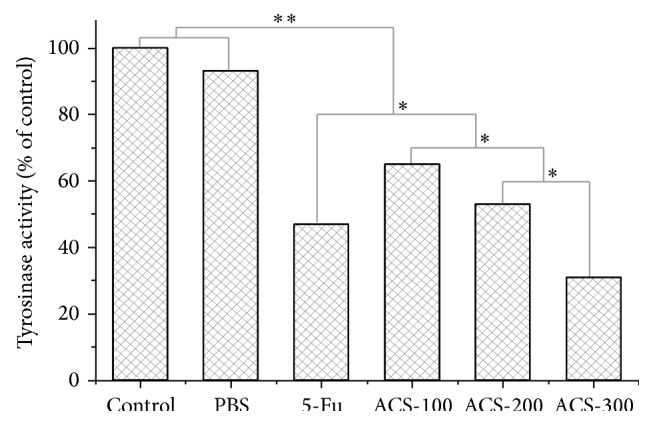
Effects of ACSs on the tyrosinase activity of B16 cells. Cells were treated with 5-Fu and various concentrations of ACSs (100, 200, and 300 *μ*g/mL), and their tyrosinase activity was determined from cellular lysates.

Almost all melanogenesis inhibitors identified thus far inhibit melanogenesis by lowering the activity of cellular tyrosinase, which plays a key role in melanogenesis. We determined the effects of ACSs on tyrosinase activity. After subjecting them to different treatments, B16 cells were lysed to obtain crude tyrosinase. We measured enzyme activity in the crude extracts using l-Dopa as an enzyme substrate, and the results are shown in Figure 7. The cellular tyrosinase activity in the crude extract significantly decreased after treatment with ACSs. ACSs directly inhibited the enzyme activity of B16 cells and restrained the formation of melanin (Figure 7).

### 3.7. ACSs Inhibited Tumor Growth and Protected the Liver and Spleen against Injury

**Figure 8:**
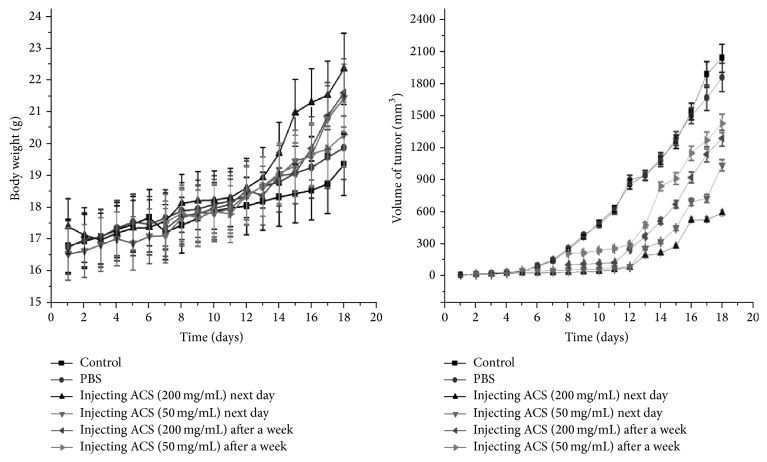
ACS-induced solid tumor growth reduction in C57 BL/6 mice injected with B16 cells. The body weights of mice were measured every day for 18 days. The tumor volume was measured using calipers and calculated with the following formula: 0.5 × long diameter × short diameter 2. Data are expressed as mean ± SEM. *P* < 0.05, significantly different from the control group.

**Figure 9:**
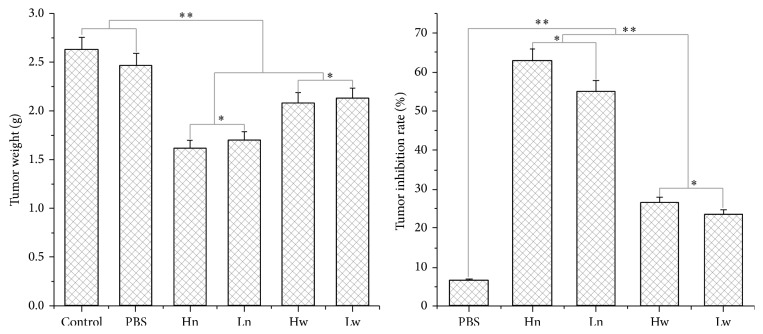
ACS-induced tumor weight reduction and tumor growth inhibition in C57 BL/6 mice injected with B16 cells. Hn: high-dose group injected with ACSs on the following day, 200 mg/mL; Ln: low-dose group injected with ACSs on the following day, 50 mg/mL; Hw: high-dose group injected with ACSs after 1 week, 200 mg/mL; Lw: low-dose group injected with ACSs after 1 week, 50 mg/mL. *P* < 0.05, significantly different from the control group.

**Figure 10:**
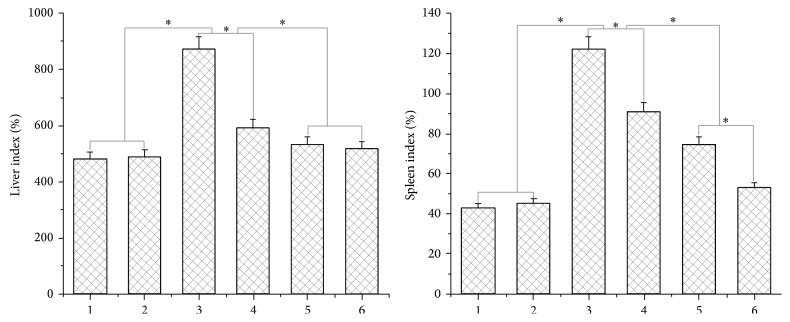
Impact of liver and spleen indices on C57 BL/6 mice injected with B16 cells. 1: without drug treatment; 2: PBS treatment; 3: high-dose ACS group, 200 mg/mL; 4: low-dose ACS group injected on the following day, 50 mg/mL; 5: high-dose ACS group injected after 1 week, 200 mg/mL; 6: low-dose ACS group injected after 1 week, 50 mg/mL. *P* < 0.05, significantly different from the control group.

ACSs had apparent inhibitory effects on the growth of melanoma. The tumor inhibition rates of treatment with high and low doses of ACSs were 62.74% and 54.94%, respectively, when ACSs were injected into the C57 BL/6 mice on the following day, whereas the corresponding tumor inhibition rates were 26.53% and 24.67%, respectively, when ACSs were induced after 1 week. Tumor development and proliferation could lead to weight reduction, pointing to the importance of early medication and demonstrating its positive effect on tumor inhibition action (Figures 8 and 9). In addition, ACSs can effectively protect the liver and spleen against injury.  Figure 10 shows that the liver and spleen indices at the ACS concentration of 200 *μ*g/mL were 8.7 and 1.22, respectively. At the ACS concentration of 50 *μ*g/mL, the liver and spleen indices were 5.9 and 0.91, respectively. When ACSs were injected after 1 week, the high- and low-dose groups exhibited liver indices of 5.25 and 5.01, respectively, and spleen indices of 0.75 and 0.53, respectively (Figure 10). These results demonstrated that ACSs better protected the liver and spleen of the mice from the high-dose group at certain ranges and suggest that early treatment of tumors can potentially protect the liver and spleen from damage.

## 4. Discussion

*A. chinense* contains various biological materials, such as sulfur compounds, steroidal saponins, nitrogen compounds, flavonoid compounds, and amino acids, among others. Saponin materials are potential anticancer agents, and different saponins possess different mechanisms of action. Their cytotoxic effects may be attributed to nonapoptotic cell death stimulation. A number of well-known processes leading to cell death exist, but they involve different mechanisms of action, such as stimulation of autophagic cell death or disassembly of cytoskeleton integrity. Saponins have been reported to inhibit cancer cell growth, invasion, and metastasis, such as HL-60 [47], human melanoma HTB-140 and human skin fibroblasts [48], human colorectal HCT-116 carcinoma [26], NCI-H460 [49], HeLa [50], and Bel-7402 [51], among others.

In recent years, an increasing number of researchers who isolated new saponins from natural sources and characterized their structures have also reported on their cytotoxic activity. Normal cell division, cellular motility, intracellular transport, and proper cell shape are all dependent on the integrity and stability of the cytoskeleton. Saponins may stimulate the disintegration of the microtubular network or actin filaments of cancer cells, which can lead to further nonapoptotic cell death [34, 52]. Historically, chemical drug therapy is the first line of cancer treatment, with the main antitumor drugs applied in clinical practice including alkylating agents, winding platinum compounds, hormonal tumors, new angiogenesis inhibitors, gene therapy, and telomerase inhibitors [53]. Among the commonly used drugs are cyclophosphamide, doxorubicin, cisplatinum, 5-Fu, and doxorubicin. Moreover, specific cancer cells not only exhibit cell toxicity and anticancer effects but also trigger several side effects on normal healthy cells. In vitro sensitivity assays and in vivo animal models are primarily used to screen and test antitumor drugs. Various in vitro sensitivity testing methods have already been established to date, including cell morphological change observation by staining cancer cells and tumor cell organelles; organizational change observation (e.g., appearance of nuclear small bodies, changes in the cytoskeleton and nuclei, mitochondrial membrane potential changes, and phospholipid membrane ectropion during cell death); cell scarification to determine the diffusion, transference, and colony formation ability of cells; and cell proliferation determination. The cell nuclei and cytoskeleton, corresponding to changes and mitochondrial organelles across the membrane potential changes observed, usually need to merge with the corresponding fluorescent dyes and stained fluorescent antibodies. Morphological changes are then observed under a fluorescent or laser scanning confocal microscope. In this study, B16 and 4T1 cells treated with 5-Fu and different concentrations of ACSs exhibited different degrees of shrinkage and did not adhere to the wall. Nuclei chromatin became pyknotic and generated several nucleosomes, the nuclei ranged from being irregularly shaped to appearing crescent, and the cytoskeleton was destroyed. ACSs significantly inhibited the proliferation of B16 and 4T1 cells. The cell growth inhibitory effect increased with an increase in the concentration of ACSs. ACSs also significantly inhibited the colony formation of B16 and 4T1 cells and reduced the tyrosinase activity of B16 cells. Therefore, the apoptosis of ACSs helps explain their inhibition of enzyme activity-related cell growth and metabolism.

Tumor metastasis is the process in which malignant cancer cells spread from the primary site to the far end of an organ [54]. Nowadays, considerable progress in surgical treatment, radiotherapy, chemical therapy, and nonsurgical treatment has been achieved, but these treatment measures do not completely prevent tumor spread and transfer. Much evidence has indicated that many patients with a malignant tumor ultimately die of cancer metastasis or tumor recurrence and that the transfer of tumor usually happens in the early stage of cancer development [55]. The spread of cancer cells and their unlimited proliferation are clearly the main factors that render cancer treatment unsuccessful. Diffusion and transfer are crucial to controlling tumor proliferation and curing patients with cancer. ACSs have an inhibitory effect on the migration ability of B16 and 4T1 cells, and the migration rates of the two cell lines exhibit a downward pattern with increasing ACS concentration.

Antitumor immunological mechanisms are very complex and involve various immune cells as well as the secretion of products, among others. Recent studies have shown that many cell factors secreted by immune cells are activated, directly or indirectly affect cancer cells, and exhibit antitumor function with immune adjustment utility. These three categories are interdependent. Active plant materials usually have a two-way adjustment function, allowing the abnormal immune state of the body to be restored without significantly affecting normal immune function. Chemotherapy and radiation therapy are primarily used for the clinical treatment of cancer, and the selection of antitumor drugs and low gamma radiation doses for animal immune models thus not only is of certain practical significance but also can reflect the targeted ACS immune adjustment effect. The spleen and thymus immune organs, such as the body's immune system, are important components of a tumor, and immune response plays an important role [56, 57]. As such, we used tumor-burdened mice for spleen index determination and found that the ACS-treated group had a higher spleen index and exhibited enhanced immunity compared with the model group. To further study the effects of ACSs on the liver index, we measured the ACS-induced changes in mouse liver weight. The experimental results showed that the liver index of the ACS-treated mice was significantly higher than that of the model group. In summary, we found that ACSs have a very good immunity enhancement effect in mice, indicating that their indirect antitumor mechanism holds potential in protecting the body against tumor tissue growth.

## 5. Conclusions

Further investigation is needed to fully examine the apoptosis of ACSs and determine their primary mechanism of action. However, the results of this study demonstrate that ACSs may represent an effective agent for melanoma and breast carcinoma therapy. Our study highlights at least four novel discoveries involving ACSs in the B16 and 4T1 cell lines. First, ACSs are cytotoxic to B16 and 4T1 cells, and they exhibit concentration-dependent and time-dependent changes. Second, ACSs decrease proliferation and affect cancer cell colony formation, ultimately to induce cell death. Third, ACSs inhibit the migration ability of B16 and 4T1 cells, with the migration rates of the two cell lines showing a downward trend as the concentration of ACSs increased. Finally, ACSs reduce the growth of mouse xenograft tumors without any apparent toxicity and have a certain degree of protective action on the spleen and liver. These findings provide a strong foundation for further evaluation of the application of ACSs for melanoma cancer therapy.
